# Why the Diagnosis of Attention Deficit Hyperactivity Disorder Matters

**DOI:** 10.3389/fpsyt.2015.00168

**Published:** 2015-11-26

**Authors:** Alaa M. Hamed, Aaron J. Kauer, Hanna E. Stevens

**Affiliations:** ^1^Child and Adolescent Psychiatry Division, Department of Psychiatry, University of Iowa Carver College of Medicine, Iowa City, IA, USA; ^2^Neuroscience Program, Pappajohn Biomedical Institute, University of Iowa, Iowa City, IA, USA

**Keywords:** attention deficit hyperactivity disorder, diagnosis, treatment, outcomes, children

## Abstract

**Background:**

Attention Deficit Hyperactivity disorder (ADHD) is one of the most common and challenging childhood neurobehavioral disorders. ADHD is known to negatively impact children, their families, and their community. About one-third to one-half of patients with ADHD will have persistent symptoms into adulthood. The prevalence in the United States is estimated at 5–11%, representing 6.4 million children nationwide. The variability in the prevalence of ADHD worldwide and within the US may be due to the wide range of factors that affect accurate assessment of children and youth. Because of these obstacles to assessment, ADHD is under-diagnosed, misdiagnosed, and undertreated.

**Objectives:**

We examined factors associated with making and receiving the diagnosis of ADHD. We sought to review the consequences of a lack of diagnosis and treatment for ADHD on children’s and adolescent’s lives and how their families and the community may be involved in these consequences.

**Methods:**

We reviewed scientific articles looking for factors that impact the identification and diagnosis of ADHD and articles that demonstrate naturalistic outcomes of diagnosis and treatment. The data bases PubMed and Google scholar were searched from the year 1995 to 2015 using the search terms “ADHD, diagnosis, outcomes.” We then reviewed abstracts and reference lists within those articles to rule out or rule in these or other articles.

**Results:**

Multiple factors have significant impact in the identification and diagnosis of ADHD including parents, healthcare providers, teachers, and aspects of the environment. Only a few studies detailed the impact of not diagnosing ADHD, with unclear consequences independent of treatment. A more significant number of studies have examined the impact of untreated ADHD. The experience around receiving a diagnosis described by individuals with ADHD provides some additional insights.

**Conclusion:**

ADHD diagnosis is influenced by perceptions of many different members of a child’s community. A lack of clear understanding of ADHD and the importance of its diagnosis and treatment still exists among many members of the community including parents, teachers, and healthcare providers. More basic and clinical research will improve methods of diagnosis and information dissemination. Even before further advancements in science, strong partnerships between clinicians and patients with ADHD may be the best way to reduce the negative impacts of this disorder.

## Introduction

Faced with controversies from the medical field, parents, and educational authorities, the diagnosis and treatment of ADHD has continued to be a source of debate (1–3). Studies have differed on their estimation for the prevalence of ADHD (4). Some have suggested that ADHD is an over-diagnosed and over-treated condition as the parent reported diagnosis of ADHD has risen from 6.9% in 1997 to 7.8% in 2003, to 9.5% in 2007, and to 11% in 2011 (5, 6). On the other hand, many child psychiatrists and healthcare providers believe that ADHD is an underdiagnosed and undertreated condition, with many cases being missed for a variety of reasons. In this review, we discuss factors that interfere with adequate diagnosis of ADHD; many aspects of a child or youth’s life contribute to missed or delayed diagnosis. We also review the consequences of a missed or delayed ADHD diagnosis. Many lines of evidence demonstrate that a failure to diagnose ADHD prevents children and their families from getting the assistance they need to achieve their full potential in academic and psychosocial settings (7). Other studies suggest that untreated behavioral problems pose significant sociocultural, academic, employment, relationship, and life coping skill deficit (8). We also review whether the presence of an ADHD diagnosis itself, irrespective of receiving treatment, has consequences. Included in this, we consider how the patient experience of receiving an ADHD diagnosis may have both negative and positive components but is certainly a critical component when considering the broader impacts of ADHD.

## Challenges Affecting Recognition and Diagnosis of ADHD

The American Academy of Pediatrics and other groups that care for children suggest that ADHD be screened for and diagnosed as early as the preschool years (9) to afford those affected the ability to achieve their full potential in school or at home (7). Many factors intrinsic to children themselves have informed these guidelines, but many factors outside of the child also play a role in how diagnosis occurs. Multiple factors may influence presentation to providers and therefore also the timing of diagnosis and treatment of ADHD including the role of parents, aspects of the medical system, school-based factors, and those intrinsic to children (10).

### Parental Role

Parents play a major role in early recognition of behavioral problems in their children which may prompt them to seek medical help (10). Parental perception of whether ADHD is a neurobehavioral disorder that requires medical management can affect their decision to seek medical help or be accepting of medical advice and treatment once evaluated. A range of beliefs about contributions to child behavior lead parents not to seek assessment for ADHD including dietary factors (11), limitations of the educational setting (12), not perceiving child behavior as a burden (1), and having a higher threshold for behavioral tolerance before seeking assessment (13). Factors other than conceptualizations about the origin of behavioral differences may also influence parents in whether and when they seek ADHD assessment. The motivation and ability to seek assessment may be affected by psychosocial stressors like financial difficulties (10), having other children, and whether their child has inattentive ADHD, which can be perceived by parents as less problematic (1). In the US, parental income and type of health insurance can strongly influence the timing of the diagnosis of ADHD (10, 14) as the use of specialist services for evaluations of their child can be a financial burden (1). Parents’ own life course may also play a role in their perception of ADHD as a problem that needs assessment (15). A study of 324 children who participated in the Multimodal Treatment Study of Children with ADHD (MTA) determined that agreement and disagreement in ratings of mothers and fathers were attributable to parental stress (16). This may represent an important factor in why ADHD comes to attention in some children and not in others, but is also an important factor for clinicians to consider when collecting data from parents about symptomology in their child.

Parental exposure to negative information about ADHD, usually from non-healthcare professionals or social media (17), can also delay the process of the diagnosis and treatment of ADHD (18). Some parents report that they believe that ADHD diagnosis is a means to “create business” for private practice physicians and/or that companies generate revenue from unnecessary use of pharmacologic treatments (13). Such findings speak to the need for community and parental education regarding the symptoms of and help available for ADHD in attempts to increase the timely diagnosis and treatment of ADHD. Higher rates of parents describing ADHD symptoms in medical terms have been associated with increased receipt of medical service (1).

Parents from different cultural and ethnical backgrounds may have different views and perceptions of behavioral norms and when to consider behavior inappropriate or indicative of a clinical disorder such as ADHD (19). The rate of ADHD in the US pediatric population based on parental report has been found to be lower in Hispanics and African Americans than in Caucasians (2, 20) and the prevalence among Hispanics were half that of non-Hispanics (6). An awareness of differing cultural beliefs and belief systems on the part of health care professionals is crucial to unify parental beliefs, clinical psychoeducation, and treatment recommendations (11). Given the diverse population of children in the US (19), different interpretations or explanations for the symptoms of ADHD exist among different cultural backgrounds (11, 21). In many immigrant communities, a psychiatric diagnosis of ADHD is associated with isolation and social exclusion (19). In some cultures, hyperactivity and/or impulsivity in boys can be endorsed as typical by parents and viewed as gender preferred behavior (22).

The level of trust and communication between parents and teachers also has a significant impact in the early diagnosis of ADHD (12). Given that the majority of the children in the US attend kindergarten by the age of 5 years and many others attend daycares and preschools earlier; effective communication between concerned teachers and parents is an important factor in early and appropriate identification of children at risk (12). Mistrust between parents and teachers can have a negative impact on seeking an assessment for an ADHD diagnosis (11). For this and other reasons, it is important that teachers and educational authorities build strong and trusting relationships with parents. This allows information to be shared about a child’s functioning and any concerns about symptoms or impairment. Additionally, parents of children with ADHD have a higher probability of having the diagnosis themselves (23) and a lack of diagnosis in parents can lead to increased risk of family conflicts and negative parent–child interactions (23).

### Health Care Providers’/Medical Role

Access of the public, children, and their families to healthcare providers and to medical research findings are influential factors in early diagnosis and recognition of ADHD. In the United States, the limited access to mental health clinicians results in the majority of children being diagnosed and treated by primary care physicians (PCP). A study of 60 ADHD patients estimated that 77.8% of the children diagnosed were treated and followed by a PCP and 22.1% were treated and followed by a child psychiatrist (24). Limited reimbursement for specialist evaluation or mental health care is also a factor in diagnosis of ADHD, particularly for complex presentations that may require more clinician time (9). There is a marked difference in the frequency of the diagnosis between US and European clinicians with this ratio estimated to be as high as 20:1, highlighting different clinical approaches, thresholds, or medical system factors that influence the diagnosis of ADHD (25).

Even when these difficulties of the assessment process do not exist, the basic definition of ADHD may pose challenges. There are inconsistencies in the definition of ADHD as it is defined in the diagnostic and statistical manual disorders-IV-TR or DSM-5 and in the international statistical classification of disease (21, 26). The validity and accuracy of ADHD as a diagnosis is still questioned by some groups as they dispute the current evidence supporting ADHD diagnosis (3, 27). One feature of ADHD diagnosis at issue is that it may be easily biased and lacks standardization (21), which is in part due to the subjectivity in evaluating children with ADHD (28). Also, the decision about the threshold of symptoms at which to seek medical attention or at which impairment occurs may largely be open to interpretation. Research is lacking on objective ways to validate the diagnosis (3). Many studies have found that the concordance of the reports from different resources like teachers’ report and parents’ evaluations for the same child is poor (28).

However, challenges for clinical diagnosticians can be addressed by some methods. Detailed prenatal, family, and educational histories are of significant value in aiding the diagnosis of ADHD (29). When evaluating children for ADHD, questions should be asked about ADHD symptoms in siblings and parents (23). Parents of children with ADHD are four times more likely to have ADHD themselves (30). A study conducted in the United States aimed at improving the use of a planned care approach to diagnosis reported that out of 60 patients with ADHD, only 5% had documentation of standardized diagnostic instruments and 57% had no documentation of any comorbidity assessment (24). Standardized tools of assessment could go far to bolstering the accuracy of diagnosis as well as the acceptance by families and others of the clinical diagnosis procedure.

### Educators’ Role

A supportive environment is an essential part of a child’s life. Children in the US spend a significant portion of their time in school making teachers and the school structure extremely influential for typical development and for the diagnosis of ADHD.

In clinical assessment of ADHD, a teacher report of a child’s behavior is often sought. However, teacher ratings of ADHD symptoms in students may have limitations. For example, a discrepancy has been shown in the level of reports of ADHD symptoms in African American students versus same age non-black peers (2). Because of the reliance on accurate teacher ratings of child behavior, the mental health community makes a significant impact when it provides training and support for educationally based professionals in the recognition and management of children with ADHD. These individuals are often the first line of recognition due to the amount of time children spend in classrooms and the differing levels of demands that can reveal children’s need for mental health intervention (31). The attitude of schools toward ADHD and their incorporation of early intervention efforts can have a significant impact on children and parents (32). Many parents express feeling unheard by teachers and school administration when concerns were raised about symptoms prior to ADHD diagnosis (32).

### Children’s Role

Numerous factors intrinsic to a child or youth can affect their diagnosis of ADHD including, gender, age, race, socioeconomic status, and severity of symptoms (11, 33). While the true rate of ADHD in boys is higher than in girls, it is also true that detection rates, and therefore formal diagnosis, may differ by gender. Boys are more prone to the diagnosis of ADHD than girls (10, 23) as they are more likely to present with the hyperactive/impulsive or combined type of ADHD, in part due to the perception that it is more impairing (12). Clinicians are also more likely to give the diagnosis of ADHD to boys when compared to girls due to a higher degree of externalizing symptoms in males (10, 28). Girls are frequently underdiagnosed as they more likely to present with internalizing symptoms (23), which can be less apparent, such as forgetfulness and difficulty sustaining attention (28).

A thorough work up for ADHD also incorporates assessment for common comorbid symptoms/diagnosis, including anxiety, depression, oppositional defiant and conduct disorders, developmental and learning disorders or other neurodevelopmental disorders. At times somatic, physical, or syndromic features can overshadow ADHD symptoms (9, 34). However, the presence of comorbidities and their severity can also increase the likelihood of service use which may be associated with increased rates of accurate diagnosis (31). For example, girls with Turner’s syndrome (XO) are more predisposed to ADHD (35) and the presence of this syndrome may either add to or detract from ADHD diagnosis.

Given the diversity of children in the US, the background history/demographics of the child is important in making an accurate clinical assessment (19). Children with different backgrounds or exposures can present with disruptive symptoms that resemble ADHD but have a different origin such as trauma from exposure to war or a language barrier from recent immigration (19). Children living in a household where English is the primary language were four times more likely to be diagnosed with ADHD than those in which English is not the primary language (6). The psychosocial histories of children and factors contributing to behavioral phenotypes may vary considerably, and so determining etiology of behaviors associated with the diagnosis of ADHD may be complex (32).

## Objectivity of the Diagnosis and Needed Research in ADHD

There is an apparent discrepancy between the lack of objective methods for the clinical diagnosis of ADHD and recent advancements in genetic, neuroimaging, and other biological assessment methods (21). Reliable biomarkers would significantly reduce some difficulties of diagnosing ADHD but findings thus far have not demonstrated distinctive biomarkers that have high sensitivity and specificity for ADHD diagnosis.

Strong genetic contributions have been reported for ADHD (7, 21, 23). It is documented that genetic influences have also been shown to mediate the continuity of ADHD between early and middle childhood (2). Studies in model systems also suggest that genetic variations may cause dysregulation of dopamine signaling and paucity of dopamine release in response to stimuli result in hyperactivity and forgetfulness similar to the symptoms ADHD (36). Despite these important discoveries about genetic risk factors, no specific genetic finding has been identified that can aid in diagnosis.

EEG and brain imaging have been investigated as a way to support and enhance the diagnosis of ADHD and choices about treatment. In a study of 10 children, medication responders had less Theta/Alpha and more Beta activity on EEG than non-medication responders (37). However, the number of subjects was small and the findings not specific for these biomarkers as well as many others that have been investigated. The behaviors associated with ADHD clearly have biological correlates which theoretically could be demonstrated through differences in functional neuroimaging (32). While research studies have demonstrated group differences in neuroimaging outcomes between ADHD patients and controls: to date the use of the radiological imaging has been limited to research about pathophysiological mechanisms and does not have a clinical role in diagnosis (2).

Some studies have examined the role of environmental factors that may predispose an individual to ADHD and thus could be helpful as diagnostic factors or indicators of risk. For example, exposure to nicotine during human prenatal development has been linked with ADHD (38), perhaps mediated through disrupted hippocampal and overall brain development as shown in animal models (39). Smoking has also been suggested to have effects across multiple generations through maternal passage of risk for ADHD to offspring (29). Family smoking history may thus take on a larger role in ADHD diagnosis as more research elucidates these risks. Another factor sometimes considered in the diagnostic process is iron deficiency. Iron plays a substantial role in the development of the central nervous system, including the dopamine, norepinephrine, and serotonin neurotransmitter systems (40). Iron-deficiency in model systems results in changes in dopaminergic signaling (41). In a hypothesized “two-hit” type of model, iron deficiency may also interact with other disruptions of dopaminergic development resulting in ADHD-like phenotypes (42). In children, studies have also suggested that iron deficiency anemia alter monoamine neurotransmitters and CNS myelination, which may be related to ADHD (41).

Serum ferritin levels have been found to be low in children with ADHD (43). Also, brain iron level as measured by MRI was low in children with ADHD suggesting the involvement of iron deficiency in the pathophysiology of ADHD (44).

Lastly, food additives have been examined for possible roles in ADHD. Some studies have found positive associations but no causal link between hyperactivity and dietary artificial food coloring and sodium benzoate preservative (45). However, other studies have suggested no relationship between food additives and ADHD (46). Managing or alleviating the symptoms of ADHD using nutritional supplements as a strategy has also been investigated; a meta-analysis of this work demonstrates that the effects of supplementation and/or exclusion are likely small and may be restricted to patients with food sensitivities (46). Further advances in these areas of research may be relevant for both the diagnosis and treatment of ADHD.

Attention Deficit Hyperactivity disorder likely arises from multiple changes in biological, psychological, and social domains from which many etiologies of small effect, both environmental and genetic, interact and result in symptoms (47). This process likely gives rise to ADHD differently in multiple kinds of vulnerable people (27), which may continue to pose challenges to diagnosis.

There is need for more research in child psychiatry at the basic biological level, focusing on early human brain development, to help better understand the pathophysiology of ADHD and child psychiatric illnesses in general (48).

## ADHD in Adolescents and Young Adults

Adolescents and youth face challenges during transition to adulthood which can limit their abilities to obtain medical services (9, 23, 49). During adolescence and young adulthood, only 50.9 and 28.9%, respectively, of those meeting DSM IV criteria for ADHD had received any treatment in the past 3 months (23). There are multiple factors that contribute to this decline in ADHD treatment that accompanies adulthood. Physician may rely more on adolescents reporting their own symptoms at this age, as they have less supervision by their parents; however, adolescents may minimize their behavioral problems, which can lower the probability of receiving an accurate diagnosis of ADHD (9). In addition, it is very common for adolescents who received a diagnosis in childhood to discontinue medical care over time (9). Physicians should obtain previous records of adolescents to review prior ADHD symptomology and to evaluate for comorbidities including substance use disorders (SUD), depression, and anxiety (50).

As ADHD is regarded as a childhood disorder, there is much less research about ADHD in adults (51). The worldwide prevalence of ADHD in adults is estimated to be 4–5.9% in all age groups (51). One study showed that using DSM IV criteria for diagnosing adults, which mandates six hyperactive-impulsive symptoms excluded almost half of adults who were at least 1.5 SD above the population mean. DSM-5 has a lower threshold for adult diagnosis, mandating four symptoms for diagnosis, which could be a more sensitive threshold for detecting individuals with functional impairment (26, 52).

## Controversies Surrounding the Treatment of ADHD

Diagnosis with ADHD matters, in part, because it is often accompanied by recommendations for treatment. Treatment of ADHD has been a source of controversy, especially in relation to the use of psychostimulants like methylphenidate (Ritalin) and mixed amphetamine salts (Adderall) and non-stimulants, such as atomoxetine, clonidine, and guanfacine for children, each of which has its own limitations (28, 53, 54). More extensive reviews exist that discuss the thresholds for use and limitations of stimulant treatment for children with ADHD (55), only some of which will be discussed here.

Stimulants used in the treatment of ADHD are thought to act by increasing brain catecholamine levels (56) and are effective in 65–75% of children after a first stimulant trial (57). Methylphenidate blocks dopamine and norepinephrine re-uptake transporters (58) and has been used for the treatment of ADHD for many decades. Despite this long history of use, the precise prefrontal cortical and subcortical mechanisms of action are poorly understood (56) for both methylphenidate and mixed amphetamine salts, which may partially contribute to ongoing controversies about their use.

Evidence suggests there has been an increased use of stimulants in US (6, 59, 60). The prevalence of stimulant use in the United States estimated to be 4.4–6.1% (6, 61), with a reported increase of 28% from 2007 to 2011(6). Another study estimated that just over half of the US children with ADHD were treated with stimulants (6). This is attributable to multiple factors including physician prescribing practices as well as parental and child attitudes about ADHD diagnosis and treatment (6, 11, 56). More clinically related variables that effect adherence to recommended stimulant treatment include how the mechanism of action of stimulants is explained and how medication routines are simplified (18, 62). In the popular press, the increased call for early diagnosis and treatment of ADHD has been attributed at times to profit-seeking actions of pharmaceutical companies (54). Others have argued that increased treatment of children with severe ADHD has led to benefits including decreased number of hospitalizations and decreased crime rates (56). Despite observations that stimulant use is increasing, epidemiological studies at the National Health and Nutrition Examination Survey (NHANES) and the National Comorbidity Survey Adolescent Supplement (NCS-A) show that many children meeting ADHD criteria do not receive treatment (5). Studies suggest that African American and Hispanic children in particular have lower prescription filling rates than Caucasian children (2, 63). This may be a result of lower percentage of diagnosis in these populations or variations in attitudes about the treatment of ADHD.

The adverse side effects of stimulant use contribute to the ambivalence some patients and families have about treatment initiation and compliance (56). Possible side effects include insomnia, decreased appetite, headache, increased blood pressure and heart rate, symptoms of anxiety and depression, and slowing of growth with the possibility that there may be modest sustained differences from a child’s anticipated adult height if children remain on stimulants for years (28, 56, 64). Some patients may benefit from their clinicians becoming more educated about optimizing stimulant use by titrating the medication until achieving the maximum benefits (9). The development of novel treatments with different side effect profiles may also provide some resolution to concerns about adverse effects. Regardless, treatment options for ADHD will always inform perceptions of the disorder itself and its diagnosis.

Other treatment modalities in addition to pharmacotherapy include: educational modifications, behavioral treatments for parents and teachers to help children modify behavior, and cognitive enhancement interventions (65). While the evidence base for each of these differs, multiple different approaches are used widely in the treatment of ADHD. All approaches elicit a variety of individual and community opinions that in turn influence whether individuals receive an ADHD diagnosis.

## Consequences When ADHD is Not Treated

One of the major consequences of ADHD not being diagnosed is a lack of treatment. Untreated ADHD can pose a tremendous amount of psychological, financial, academic, and social burden to the individual and the community, which reflects the importance of diagnosing and treating the disorder (23, 66). While treatment has not been shown to completely “normalize” the developmental trajectory of individuals with ADHD, individuals with ADHD who do not receive treatment have poorer long-term outcomes compared to those that are treated (51). Untreated ADHD during childhood is a risk factor for later adult mental health issues, which extend beyond impairment in academics (66). A lack of treatment for ADHD also impairs social and occupational functioning and increases the likelihood of developing comorbid disorders like anxiety, depression, personality disorders, antisocial behaviors, and SUD (66, 67). Many mechanisms may be at work linking undiagnosed ADHD to vulnerabilities (27).

The consequences of untreated ADHD has been shown in many different domains of functioning.

### Academic Achievement

A longitudinal study that examined the impact of stimulant treatment in ADHD to academic outcome during late adolescence found that children treated with stimulants showed academic gains on several measures relative to children who did not receive treatment (68).

### Family/Relationship

Symptoms of hyperactivity that would typically be addressed by treatment are linked, when untreated, to long-term social and peer problems (69). Poor listening skills and poor frustration tolerance that persist in untreated ADHD can lead to tension at work and home, decreased intimate relationships, internalizing problems, and low self-esteem in adulthood (23). When ADHD persists in parenthood, there is an increased risk of family conflict and negative parent–child interactions; this may be related to parents with ADHD being easily frustrated and unable to control their emotions and impulses when untreated (23). A study based on a clinical sample also found a connection between childhood ADHD and intimate partner violence (IPV) in adulthood in association with conduct disorder (CD) and when controlling for CD as a comorbidity (70).

### Substance/Alcohol Abuse

Much work over the past decades suggests that there is a strong link between ADHD and substance/alcohol abuse (23, 66, 71). An increase in risk for substance use, nicotine addiction, and alcohol abuse was conferred by even a single symptom of hyperactivity or inattention (71). While many individuals treated for ADHD continue to have persistent symptoms, this study also suggests that the initiation of substance use was more likely with more uncontrolled symptoms. Having ADHD is associated with an earlier onset of psychoactive substance abuse across the life course (23). For adolescent boys and girls, increased levels of hyperactivity and impulsivity symptoms had a statistically significant prediction for all substance abuse, nicotine dependence and cannabis abuse, even when controlling for conduct disorder as a comorbidity (71).

Links between ADHD and SUD may be bidirectional (66). Adults with SUD have been shown to have a higher rate of ADHD than the general population and, in this population, ADHD contributes to increased rates of comorbid disorders, previous criminal convictions and suicide attempts (66). Substance using individuals with ADHD compared to those with only SUD had higher self-rated impairment across several domains of daily life and higher rate of substance abuse and alcohol consumption, suicide attempts, and depression. These findings highlight the negative impact of ADHD on individuals and the increased burden they place on the mental healthcare system (66).

### Financial/Employment

Attention Deficit Hyperactivity disorder, especially when untreated, can lead to financial and employment difficulties (8, 33, 72). Studies suggest that uncontrolled ADHD can negatively affect work efficiency leading to longer hours of work, job instability, lack of insight into future goals, and lower annual personal incomes (23, 33). Across 10 national surveys examined in the WHO World Mental Health (WMH) Survey Initiative, workers with ADHD had significantly more sick days per year and higher annualized average excess numbers of workdays associated with reduced work quantity and quality (72). Even if some adults are able to maintain appearances, overcoming impairments that are not treated may be a struggle that can interfere with general life satisfaction (23, 73). Procrastination, distractibility, frequent lost workdays, and disability have high economic burden with greater utilization of medical services (23, 74).

### Criminal

Untreated ADHD is associated with higher rates of criminality, imprisonment, speeding, traffic violation, and motor vehicle accidents (23, 75, 76).

### Comorbidity

Longitudinal research that followed hyperactive children for 10 years has suggested that hyperactivity is a risk factor for increases in other psychiatric diagnosis (69). The precise relationship between CD/ODD and ADHD, especially the mechanism of development of antisocial behavior in children with ADHD, is not fully understood. However, aggressive behavior which occurs in some children with ADHD, particularly without treatment, is highly predictive of whether juvenile offenders will reoffend (77).

### Mortality

Mortality rate in a recent study of an ADHD population was 5.85 per 10,000 person-years compared with 2.21 in controls. Interestingly, the highest rate of mortality was found in people diagnosed in adulthood (78). When diagnosed between 1 and 5 years, the mortality rate ratio (MRR) for individuals with ADHD was 3.34%. For diagnosis between ages 6 and 17, the MRR increased to 4.34%, and if ADHD was diagnosed after 17 years of age, the MRR is 22.28% (78). This emphasizes the importance of early diagnosis and treatment in ADHD. Children with ADHD, adolescents in particular, have a significantly higher suicide rate at three times higher than the national average. While no evidence supports a direct link between untreated ADHD and suicide, the increased risk may be conferred by ADHD comorbidities (2). Comorbidities like oppositional defiant disorder, conduct disorder, and SUD increase the MRR in ADHD and when adjusted for these comorbidities, ADHD remains associated with increased MRR (78).

## Consequences When ADHD is Not Diagnosed

Many of these issues surrounding perception of ADHD and the ability to effectively assess individuals for ADHD influence whether or not accurate diagnoses of ADHD are made. While there are significant data detailing the consequences of ADHD not being treated, fewer studies have been performed to understand whether diagnosis matters independently. ADHD diagnosis and its associated opportunity for psychoeducation and prognostic information may have important effects on development.

The few studies that have been done suggest that there are consequences in a variety of domains. First, evidence suggests lower levels of educational attainment in individuals with ADHD who are undiagnosed compared to those diagnosed (8, 33, 68). The prevalence of undiagnosed ADHD within a substance treatment population was approximately fivefold higher than the general population (66) suggesting that undiagnosed ADHD may have substance abuse requiring treatment as a consequence. The relative underdiagnosis of ADHD in girls may lead to more women gaining an ADHD or other diagnosis in adulthood, with high rates of inattentive or impulsive symptoms at times diagnosed as depression and borderline personality disorder (23). Undiagnosed ADHD subjects are less likely to report that a first-degree family member had been diagnosed with ADHD; missed diagnosis in parents or sibling can result in missed or delay diagnosis in other members of the family (33). Also, as reviewed previously, the MRRs of individuals with delayed diagnosis of ADHD has been shown to be significantly higher than those diagnosed earlier, suggesting that a lack of diagnosis may accumulate risks to mortality (78).

## The Experience of Having the Diagnosis of ADHD

Several studies have focused on examining the experience described by individuals with ADHD. Understanding the emotional and psychological burden of this diagnosis and its impact in a child or youth’s quality of life and well-being may help to inform controversies about diagnosis and the basic perception of ADHD.

Having ADHD has been shown in multiple studies to be a source of stress, with some individuals recalling a constellation of feelings of loneliness, isolation, low self-esteem, and struggle to get along with their parents and peers at school (32, 79, 80). Despite struggles related to ADHD symptoms, receiving the diagnosis does not always reduce stress for an individual. Some individuals have described perceiving a certain degree of stigma attached to being diagnosed with ADHD, influencing their willingness to disclose their diagnosis to other people. Additionally, others have reported feeling that ADHD is viewed by others as a “convenient excuse” for their behavioral problems and they may be labeled as a “problem person” rather than a person who has a problem (79, 80). At times, ADHD diagnosis led young people to feel hurt by their peers when they were teased or targeted because of their apparent academic delay and labeled as “retarded” (79). Some perceived their identity was challenged by a diagnosis of ADHD and also felt less in-control of their lives, especially when faced with the prospect of taking medication for life (32). Still other individuals report having the diagnosis of ADHD lead them to feel as though they were different and isolated (79).

On the other hand, some individuals have described that the extra attention from their teachers or parents that accompanied the diagnosis of ADHD helped affirm and build confidence (79, 80). A qualitative study in which participants described their experience in their own words classified the psychological impact of receiving the diagnosis of ADHD into different emotional stages of psychological acceptance including both positive and negative aspects: (a) relief and optimism, (b) denial, (c) anger and resentment, (d) grief, (e) mobilization, and (f) accommodation (32). Individuals diagnosed late reported low self-esteem and underachievement due to repetitive experiences of failure during childhood (80). Clearly, ADHD diagnosis during childhood or adolescence has mixed consequences psychologically for young people.

## Conclusion

We have reviewed many of the complexities that lead to and arise from the diagnosis of ADHD. One of the key conclusions from this body of literature is that receiving the diagnosis of ADHD may benefit individuals by creating the possibility for treatment. A significant body of research has demonstrated that treatment is strongly associated with improved outcomes. Outcomes after receiving an ADHD diagnosis independent of treatment are not well understood; however, significant psychological effects of receiving a diagnosis have been described. These latter findings are in line with the complex experience clinicians have as they partner with children, youth and families coping with behavioral problems. It is precisely this partnership that then takes on greater significance when faced with such a double-edged sword in diagnosing and treating ADHD. Medical knowledge about the benefits of treating ADHD, together with the concern that clinicians feel for their patients’ long-term success, will hopefully reduce barriers to diagnosis in the future. This may be accomplished through psychoeducation, greater communication between clinicians, parents, and educators, and more research on biomarkers. Although barriers exist, clinicians who truly partner with their patients also understand that individuals receiving an ADHD diagnosis may experience immediate and complex emotions as well as challenges to their identity. The process of diagnosing ADHD is not unlike the diagnosis of other chronic and serious medical conditions and thus can benefit positively from the medical perspective. This perspective has the potential to positively impact the entire life course of individuals with ADHD as well as their families. The partnership between clinician and patient is one of the most important ways in which this benefit can arise.

## Author Contributions

AH and HS conceived, designed, acquired background material, and drafted this work; AH, AK, and HS interpreted the background material, critically revised, and approved the final version of and agreed to be accountable for this work.

## Conflict of Interest Statement

The authors declare that this work was completed in the absence of any commercial or financial relationships that could be construed as a potential conflict of interest.
